# Breaking the silence and building strength; rethinking menopause care through exercise and cultural insight

**DOI:** 10.3389/fgwh.2026.1683735

**Published:** 2026-02-16

**Authors:** Gayathri Delanerolle, Vindya Pathiraja, Sohier Elneil, Om Kurmi, Vikram Talaulikar, Paula Briggs, Lucky Saraswat, Helen Felicity Kemp, Yassine Bouchareb, Cristina Laguna Benetti-Pinto, Tharanga Mudalige, Nirmala Rathnayake, Abirame Sivakumar, Fred Tweneboah-Koduah, Nana Afful-Mintah, Nihal Al-Riyami, Lamya Al-Kharusi, Jian Qing Shi, George Uchenna Eleje, David Ikwuka, Pradip Mitra, Bernard Mbwele, Rabia Kareem, Mohammad Irfan, Peter Phiri

**Affiliations:** 1 Institute of Applied Research, University of Birmingham, Birmingham, United Kingdom; 2 School of Medicine, Hampshire and Isle of Wight Healthcare NHS Foundation Trust, Southampton, United Kingdom; 3 Faculty of Allied Health Sciences, University of Ruhuna, Matara, Sri Lanka; 4 Institute of Womens Health, University College London, London, United Kingdom; 5 Reproductive Medicine Unit, University College London Hospitals NHS Foundation Trust, London, United Kingdom; 6 Faculty of Health and Life Science, Coventry University, Coventry, United Kingdom; 7 Department of Obstetrics and Gynaecology, Liverpool Women’s Hospital NHS Foundation Trust, Liverpool, United Kingdom; 8 Department of Gynaecology, University of Aberdeen, Scotland, United Kingdom; 9 Trauma Healing Together, Aberdeen, Scotland, United Kingdom; 10 Department of Obstetrics & Gynecology, Sultan Qaboos University Hospital, Muscat, Oman; 11 Department of Tocogynecology, University of Campinas, UNICAMP, Campinas, Brazil; 12 Department of Obstetrics and Gynaecology, University of Jaffna, Jaffna, Sri Lanka; 13 Obstetrics & Gynecology, Narh-Bita Hospital, Tema, Ghana; 14 Obstetrics & Gynecology, Ethos Clinic, Milton Keynes, United Kingdom; 15 Department of Statistics and Data Science, Southern University of Science and Technology, Shenzhen, China; 16 Obstetrics & Gynecology, Nnamdi Azikiwe University, Awka, Nigeria; 17 School of Medicine & Pharmacy, University of Rwanda, Kigali, Rwanda; 18 Endometriosis Society, Kolkata, India; 19 Department of Epidemiology, Biostatistics and Clinical Research, University of Dar es Salaam, Dar es Salaam Region, Tanzania; 20 Peshawar Medical College, Riphah International University, Islamabad, Pakistan; 21 Department of Applied Psychology, University of Southampton, Southampton, United Kingdom

**Keywords:** care, cultural insight, exercise, menopause, rethinking

## Abstract

Menopause remains a largely neglected aspect of women's health in many low- and middle-income countries (LMICs), particularly across Asia, Africa, and the Middle East. Despite the profound physical, cognitive, and emotional changes it entails and the long-term health implications, access to menopause care is limited, and cultural taboos often prevent open discussion and timely support. This article explores the critical role of exercise as a cost-effective, sustainable, and culturally adaptable intervention for managing menopausal symptoms, including vasomotor disturbances, depression, anxiety, cognitive decline, and sleep disruption. Drawing on evidence from neuroscience, public health, and sociocultural research, it highlights the neurochemical benefits of physical activity, such as mood regulation and improved brain function. It also critically examines how religious beliefs, social norms, gender roles, and policy gaps influence women's ability to engage in exercise across different cultural settings. Community-based programmes, corporate initiatives, and digital adaptations underscore pragmatic approaches to integrating exercise into menopause care. We call for healthcare systems, policymakers, and researchers to address systemic neglect, normalise menopause discourse, and embed culturally sensitive, movement-based interventions into broader women's health strategies.

## Background

Menopause is a significant life stage for women worldwide, although its management is often overlooked in health agendas beyond reproductive age (1). By the late 2020s, an estimated 76% of postmenopausal women globally will live in developing regions across Asia, Africa, and the Middle East (2). It is widely recognised as a physiological transition associated with symptom variability, where some individuals experience minimal disruption while others experience severe and acute manifestations (e.g. vasomotor symptoms), alongside longer-term changes in cardiometabolic, musculoskeletal, and neurocognitive risk profiles (3). These experiences are heterogeneous and non-universal (4). Menopause is characterised by symptom patterns and health risks that unfold across distinct temporal trajectories, underscoring the need for stage-specific understanding and intervention. Acute symptoms typically emerge during the perimenopausal transition and early postmenopause and include vasomotor disturbances, sleep disruption, mood lability, and somatic discomfort, which may fluctuate in intensity but can substantially impair daily functioning (5). Persistent symptoms, such as genitourinary syndrome of menopause, chronic pain, fatigue, and ongoing psychological distress, often continue well beyond the menopausal transition and may require sustained management strategies. In contrast, longer-term risk trajectories develop more gradually and include increased risks of cardiovascular disease, metabolic dysfunction, osteoporosis, sarcopenia, and cognitive decline, reflecting cumulative hormonal, metabolic, and lifestyle influences (6). Distinguishing between these temporal domains is essential for aligning prevention, symptom management, and long-term health promotion strategies throughout midlife and beyond. Additionally, access to appropriate care remains highly uneven, especially in low- and middle-income countries (LMICs), where healthcare professionals with specialised expertise in menopause are often lacking, making menopause a pressing global public health issue (7, 8).

**Figure 1:**
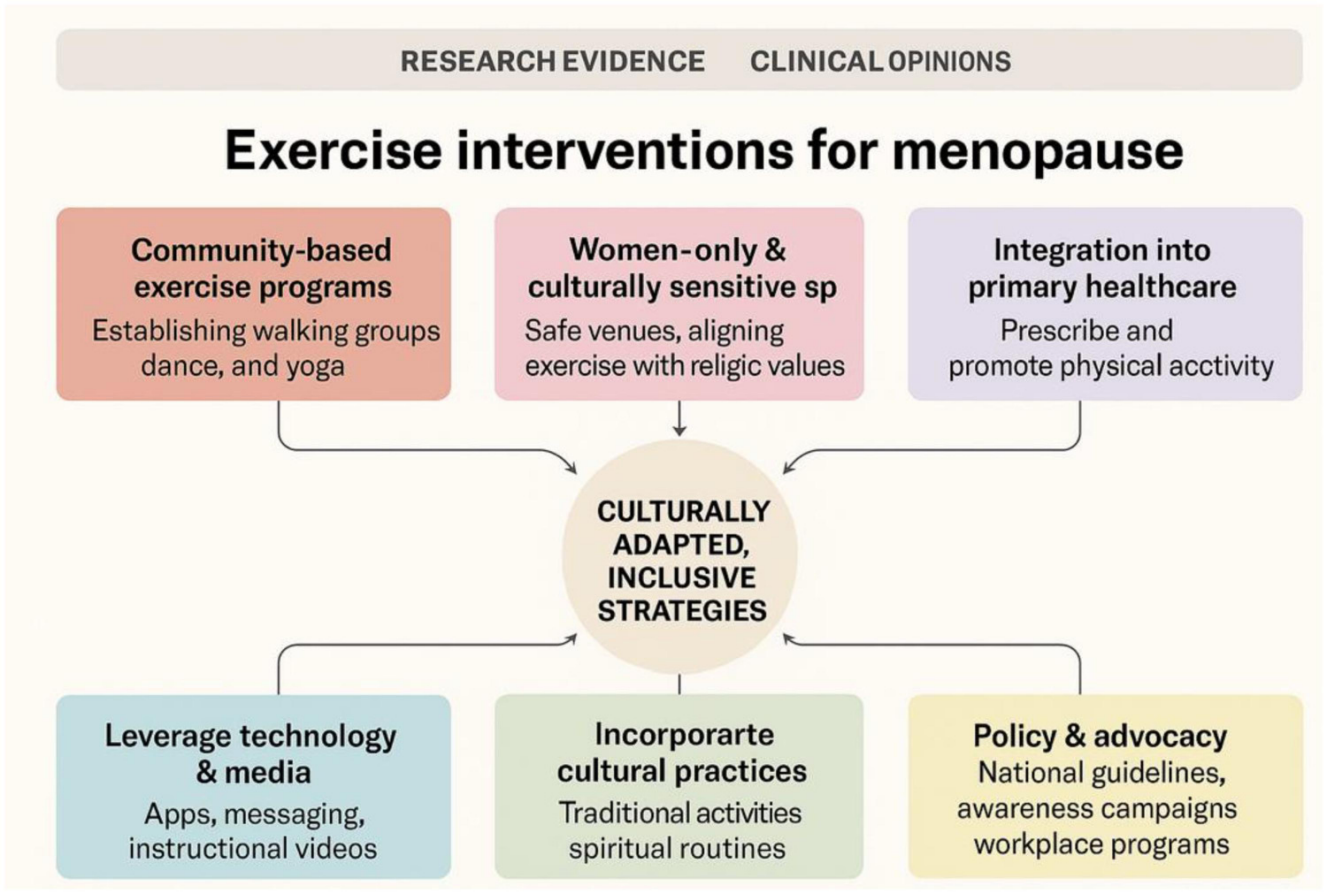
A visual of the framework MARIE exercise framework that is proposed to be used to develop interventions for people experiencing perimenopausal, menopausal, and postmenopausal.

Despite being the most effective intervention for menopause-related conditions, hormone replacement therapy remains underutilised in LMICs due to barriers such as high costs and limited access (9, 10). Exercise has emerged as a promising, cost-effective strategy to reduce the severity of menopausal symptoms and improve quality of life (1). Beyond physical benefits, regular exercise can positively impact brain health by modulating mood and neurochemistry. This article critically examines the role of exercise in managing menopausal symptoms for women in Asia, Africa, and the Middle East, drawing on evidence from research and reports. It also explores how cultural and religious beliefs influence women's access to exercise, assesses current policy landscapes and healthcare system gaps, and highlights case examples. Finally, we propose pragmatic, sustainable approaches for integrating exercise into menopause care in culturally appropriate ways, encompassing natural, surgical, and medical menopause from perimenopause to postmenopause.

### Physical activity vs. exercise

Physical activity refers to any bodily movement produced by skeletal muscles that results in energy expenditure and includes incidental activities (such as household tasks or active transport), occupational activity (work-related physical demands), and lifestyle movement (walking, gardening, or caregiving) (11). In contrast, exercise is a subset of physical activity that is structured, planned, intentional, and repetitive, undertaken with the specific objective of improving or maintaining physical fitness, health, or functional capacity (12). Both physical activity and exercise can be strategically leveraged to support menopausal health, but their feasibility and acceptability vary across health systems and cultural contexts (13). In resource-limited or time-constrained settings, integrating incidental and lifestyle physical activity into daily routines may offer a more accessible and culturally congruent approach, whereas structured exercise programmes may be more feasible within formal healthcare systems or organised community settings. Recognising and valuing both forms enables the development of equitable, context-sensitive strategies to promote physical and mental well-being during the menopausal transition.

### Benefits of exercise for menopausal symptom management

Exercise should be conceptualised as a multisystem, non-pharmacological intervention with broad relevance across the menopausal transition. Regular participation in appropriately prescribed exercise has been shown to alleviate vasomotor and somatic symptoms, including hot flushes, sleep disturbance, fatigue, and musculoskeletal discomfort, through improvements in thermoregulatory stability, autonomic balance, and inflammatory modulation. Beyond somatic effects, exercise confers substantial benefits for neurocognitive and psychological health, supporting mood regulation, stress resilience, sleep quality, and cognitive function via neuroendocrine, vascular, and neuroplastic mechanisms. Additionally, exercise plays a critical role in preserving cardiometabolic and musculoskeletal health, mitigating menopause-associated increases in cardiovascular risk, insulin resistance, central adiposity, sarcopenia, and bone loss. By acting simultaneously on hormonal, neural, metabolic, and structural pathways, exercise offers a holistic, scalable, and adaptable strategy for improving health-related quality of life in menopausal individuals across diverse clinical and cultural contexts.

Regular physical activity is a well-established non-pharmacological intervention that confers wide-ranging benefits for midlife and older women. Delanerolle et al.’s (14) meta-analysis indicated cardiometabolic disorders could be reduced by the use of hormonal-replacement therapies (HRTs), although the data for the primary studies were primarily from high-income countries (HICs). Similarly, the risks of exacerbation of menopausal symptoms need to be considered within this context. One such issue could be pelvic organ prolapse, or persistent stress incontinence and urinary tract infections, as indicated by Mudalige et al. (15) who showed the lack of menopause even being considered as part of the studies that they conducted a meta-analysis on.

For menopausal women, exercise can alleviate many symptoms and health risks (Table 1) by releasing a host of mood-boosting neurochemicals. When women exercise, the body increases production of endorphins, serotonin, and dopamine neurotransmitters that promote euphoria and combat stress and depression. This makes exercise a valuable adjunct to menopause care for maintaining not just physical fitness but also “neuro-fitness” (1, 16).

**Table 1:** Building strength through movement: evidence-based exercise benefits across menopausal symptom domains.

Symptom theme	Description	Evidence
Mood and brain health	Menopause often brings mood swings, anxiety, “brain fog”, and even depression due to hormonal changes	A 2024 meta-analysis of 26 trials found that exercise had a significant antidepressant effect in postmenopausal women (pooled effect size SMD ≈ −0.71) and also reduced anxiety (17).
		Mind–body exercises such as yoga and tai chi were especially effective, yielding the greatest improvements in depression scores
Emotional well-being	Psychological benefits translate to improved emotional stability, better sleep, and sharper cognitive function	Research on menopausal brain health suggests that maintaining an active lifestyle—alongside diet and stress management—helps the brain “recalibrate” and may buffer against memory issues (18).
		Physical exercise is even considered a protective factor against long-term cognitive decline and dementia in postmenopausal women (16). By enhancing cerebral blood flow and stimulating neural pathways, exercise supports brain plasticity during the menopausal transition
Vasomotor and sleep	While evidence on exercise reducing hot flashes is mixed, staying active can help manage triggers and improve overall tolerance of vasomotor symptoms. Notably, exercise can mimic some effects of menopausal hormone therapy by increasing endorphin levels	Furthermore, exercise is linked to better sleep quality. A recent systematic review showed mind–body exercises significantly improved sleep in peri- and postmenopausal women (SMD ∼ −0.48) (1).
Bone and cardiovascular health	Postmenopausal women face a higher risk of osteoporosis and cardiovascular disease due to oestrogen loss. Regular weight-bearing and aerobic exercise mitigates these risks by preserving bone mineral density and improving heart health	A meta-analysis found mind–body exercise increased bone density (SMD 0.41) in menopausal women (1). Exercise also helps manage weight and blood pressure, thereby reducing cardiovascular strain. In summation, an active lifestyle addresses multiple menopausal concerns—from joint aches to metabolic changes—contributing to overall well-being (19). There are studies that even the sexual and urinary symptoms also improve with exercises.
		Guided pelvic floor physical therapy: Strengthening pelvic floor muscles through guided physical therapy can alleviate urinary symptoms. Tailored exercise programmes, often incorporating Kegel exercises, are central to pelvic health management (20).
		Pelvic floor exercises: Pelvic floor muscle training (PFMT) has been associated with improvements in sexual function among postmenopausal women. A systematic review found that PFMT was the most commonly recommended exercise, showing significant benefits for sexual function (21)
		Tai chi and yoga also help to enhance some symptoms (22).

### Asia: cultural beliefs, access to exercise, and policy gaps

In Asia, diverse cultural and religious contexts shape women's experiences of menopause and their attitudes towards exercise. Many Asian societies traditionally view menopause as a natural life transition rather than a medical condition, which can be a double-edged sword. On the one hand, there may be less overt pathologising of menopausal symptoms; on the other, women may feel expected to endure symptoms quietly without seeking help.

In many LMIC contexts, attitudes towards menopause and attitudes towards exercise are deeply interconnected, particularly for midlife women. Cultural norms that position menopause as a private experience to be endured silently often coexist with restrictions on women's bodily autonomy, gendered expectations surrounding aging and physical visibility, and social norms that limit women's participation in public or structured physical activity. These intersecting cultural and social constraints directly shape the acceptability, feasibility, and uptake of exercise during midlife. Treating attitudes towards menopause and exercise as independent constructs reflects a Global North conceptual separation that does not adequately capture the lived realities of women in many LMIC settings, where health behaviours are embedded within broader gendered, cultural, and structural contexts.

Cultural norms and gender roles in parts of Asia have historically deprioritised leisure exercise for midlife women (23). For example, Confucian-influenced cultures emphasise women's duties to family and valorise intellectual pursuits over physical activity. In a study of Asian American midlife women reflecting on their heritage, the participants felt that “physical activity was perceived to be *not for Asian girls*” because traditional values didn’t place importance on women exercising (24). Women often put household responsibilities and care of children first, leaving little time for themselves. Additionally, strong notions of modesty and propriety can limit the appropriate activities for women. In South Asian communities, many women are nervous about participating in exercise because certain activities conflict with social expectations of modesty and femininity. High-impact or gym-based exercises, for instance, may be seen as unseemly or too exposing. As a result, Asian women may opt for walking or home-based routines over public sports. One qualitative insight noted that South Asian midlife women would benefit from more culturally tailored support, providing women-only exercise spaces, guidance on suitable exercise forms (e.g. yoga and dance), and community awareness to normalise women's fitness (25).

Despite these barriers, traditional Asian practices and perspectives can also facilitate exercise. Yoga, tai chi, qigong, and other mind–body exercises have their origins in Asia and are widely respected (26). These gentle forms of activity are often culturally acceptable for older women and have proven benefits for menopause management. For instance, yoga and tai chi classes for middle-aged women have gained popularity in countries such as India and China, blending exercise with cultural wellness philosophies. Some Asian cultures frame menopause in a more positive light, such as the Japanese term *konenki* which implies a period of renewal and regeneration. In Japan, where diet and lifelong physical activity are emphasised, women historically reported fewer hot flashes and attributed their smoother midlife transition to a healthy lifestyle. Researchers have credited Japanese women's relatively late menopause to a combination of diet, regular exercise, universal education, and preventive healthcare traditions. This suggests that integrating exercise into daily life, common in some East Asian contexts, can yield tangible benefits (16).

### Healthcare system challenges and policy gaps in Asia

Across much of Asia, menopause care has not received the policy attention it deserves (27). Health systems in low- and middle-income Asian countries traditionally focus on maternal and child health, with limited resources dedicated to older women's health. As an example, India's national health programmes have long centred on family planning and safe childbirth, resulting in menopausal health being sidelined. There is a pronounced data and research gap on menopausal women in Asia, which hampers evidence-based policy development.

Consequently, few countries have comprehensive guidelines or public initiatives addressing menopause or promoting exercise for midlife women. Where clinical guidelines exist (e.g. the Indian Menopause Society's recommendation), implementation remains limited (28). Social stigma also plays a role: many Asian women hesitate to discuss menopause openly, so they may not seek out exercise programmes even if available (29). The lack of targeted interventions means women often rely on self-care. Notably, Asian women aware of exercise benefits treat it as self-care; a study in Saudi Arabia (West Asia) found that participants viewed exercise as a “valuable self-care practice” during menopause and sought out home exercise videos for guidance. This points to an unmet need for supportive infrastructure. Overall, the Asian context calls for greater policy recognition of menopause as a health priority and culturally sensitive programmes that encourage exercise among midlife women.

### Africa: sociocultural beliefs, stigma, and systemic challenges

African women experience menopause against a backdrop of varied cultural beliefs—ranging from reverence for older women's wisdom to stigma and misconceptions. In many parts of Africa, menopause has traditionally been a private matter, not openly discussed, which affects how women cope and whether they engage in health-seeking behaviours such as exercise (30). As activist Sue Mbaya noted, “negative cultural beliefs about menopause” in some African communities fuel stigma, with menopausal women unfairly deemed “unattractive” or “incapable”. This stigma can isolate women and discourage them from participating in public activities, including exercise groups. Indeed, qualitative studies in countries like Zimbabwe and South Africa revealed that many women had received little information about menopause and felt they simply had to “endure” the physical and psychological symptoms in silence (31). Such attitudes reflect a gap in education and support. However, Africa is culturally diverse, and positive perspectives upon which to build. In numerous African societies, postmenopausal women attain *greater social freedom and authority*, no longer bound by certain reproductive-related restrictions. For example, a woman beyond childbearing age may enjoy more respect in some Islamic African communities and parts of sub-Saharan Africa. She can take on leadership roles within the family or community (32).

Anthropological accounts from West Africa describe “menopausal matriarchs” who become key decision-makers and custodians of knowledge in their communities. This elevated status could be leveraged to engage older women in community wellness initiatives, as they may influence and mentor younger women. Physical activity patterns in Africa are also shaped by lifestyle and beauty ideals. In rural areas, women's daily lives often involve substantial physical labour (farming, fetching water, etc.), which can maintain fitness but is not usually framed as exercise. In urban settings, more sedentary lifestyles prevail, yet formal exercise is not widespread, especially among older women. Social determinants play a role: one study noted that in Ghana, cultural perceptions of body image influence postmenopausal women's activity levels. If a fuller figure is associated with status or health, women might be less motivated to exercise for weight control, highlighting the need to tailor messages about fitness in culturally relevant terms. Moreover, common barriers such as time constraints and a lack of facilities are pronounced for African women. Women often prioritise family needs and may have limited leisure time or safe spaces to exercise (33).

### Healthcare and policy context in Africa

Menopause has only recently started to gain visibility on the policy radar in Africa. Most African health systems face competing urgent issues (infectious diseases, maternal mortality, etc.), and menopause care has been largely neglected. As a result, there is minimal government programming for menopause; few clinics specialise in midlife women's health, and healthcare providers may receive little training on managing menopause beyond offering menopausal hormone therapy if available. This lack of structured support means that interventions such as exercise are not systematically promoted. The situation is beginning to change: grassroots movements and NGOs are spearheading a “menopause revolution” in parts of Africa. They emphasise awareness, destigmatisation, and lifestyle management. In countries such as South Africa and Uganda, new menopause societies and support networks are forming. However, large gaps remain—especially in rural areas where information is scarce and in health policies that rarely mention menopause. The need for context-specific research is acute; as Mbaya observes, sub-Saharan Africa suffers from “low levels of development, competing needs and lower investment in research” on menopause (34).

Addressing these gaps will require including menopause in national health strategies and recognising that simple lifestyle interventions such as exercise can have outsized benefits for this population of women who are living longer than ever before.

### Middle East: traditions, taboos, and opportunities for exercise

In the Middle East (including North Africa and West Asia), women's access to exercise during menopause is influenced by conservative social norms and religious practices. Many countries in this region have strong traditions around gender roles and modesty, which can create specific challenges for women's physical activity. For example, gender segregation and dress codes in conservative societies mean women often need women-only spaces or appropriate attire to exercise comfortably. A systematic overview of 17 Middle East and North Africa (35) countries identified *gender and cultural norms* as among the most commonly reported barriers to physical activity for women (36). Simply put, being female and of advanced age in these societies is associated with less exercise, in part because older women are expected to remain home or are not encouraged to engage in sport. Practical hurdles such as a lack of female gyms, limited time due to family duties, and even harsh climate (extreme heat) further compound the issue. Cultural and religious beliefs can both hinder and help menopausal women seeking exercise. On one side, menopause remains a sensitive or even taboo topic in parts of the Middle East. In Saudi Arabia, for instance, many women silently endure menopausal changes due to cultural taboos and fear of stigma. This silence can prevent them from seeking group support or asking doctors about non-pharmacological strategies such as exercise. Interviews with Saudi women revealed concerns about being seen as “old” or less attractive, leading them to keep symptoms private.

On the other side, Islamic teachings provide an opportunity: after menopause, women are relieved from certain religious restrictions (such as fasting during menstruation or observing strict purdah in some interpretations), potentially giving them more freedom to engage in activities outside the home. Many Middle Eastern women view menopause positively as it grants a “relief from menstruation and a newfound freedom to engage in religious activities at any time” (37). This positive outlook can be harnessed to encourage postmenopausal women to invest in their health. Indeed, some women in the region are proactively adopting exercise as self-care. The Saudi qualitative study noted that participants embraced holistic health practices—maintaining a balanced diet, regular exercise, meditation, and good sleep—to cope with menopause, often preferring these to medical treatments.

### Policy and health system gaps in the Middle East

Much like Asia and Africa, the Middle East has only nascent recognition of menopause in health policy. Few Middle Eastern countries have national guidelines or public education campaigns on menopause (38). Women in conservative Arab states may have limited access to specialised care; for example, discussion of menopausal hormone therapy or menopause management might be minimal during routine clinic visits.

Healthcare providers may not be fully trained to address menopause beyond treating it as a natural stage. In the Saudi study, women reported that doctors seldom brought up menopause management proactively—one noted that her gynaecologist “did not discuss anything about hot flashes or hormonal treatments”, focusing only on issues like screening and pelvic floor exercises (39). This indicates a gap in provider engagement and patient counselling. On a policy level, some countries are beginning to include women's health across the life course in their strategic plans, but implementation is slow. The lack of public conversation is a key issue; normalising menopause in the Middle East will require breaking the taboo so that women feel comfortable joining exercise classes or advocacy groups. Encouragingly, there are early signs of change—for instance, menopause was brought to the floor of Ghana's parliament by a politician and is being included in feminist agendas in Africa, and similar advocacy could spread to Middle Eastern contexts through women's health NGOs or influential figures.

### Case studies: community initiatives and emerging programs

Despite challenges, innovative programmes in all three regions demonstrate how exercise can be woven into menopause support with culturally sensitive approaches (Table 2) using examples offering learning points. They show that culturally aligned approaches, whether leveraging community solidarity in Africa, workplace infrastructure in Asia, or digital connectivity in the Middle East, can successfully integrate exercise into menopause care.

**Table 2:** Examples for community initiatives and emerging programmes.

Programme	Location	Discussion
Meno-fitness	Zimbabwe	A standout example is the “Let's Talk Menopause” initiative in Zimbabwe, founded by activist Primrose Hove. This community group has over 4,000 women, including a “meno-fitness” sub-group where >500 women exercise daily in groups
Workplace menopause support	India	A novel development is occurring in the corporate sector. Large companies in India (e.g. HSBC India, IBM, and Hindustan Unilever) have begun menopause support programmes for employees, breaking the cultural taboo in workplaces. These initiatives typically include awareness workshops, flexible work policies, and coverage for menopause-related healthcare. Some also encourage healthy lifestyles—offering sessions on nutrition and exercise to help women manage symptoms naturally. While not community-based, these programmes provide a model for institutional support. By acknowledging menopause and providing resources (such as yoga or fitness classes at work), employers are filling a gap in health education. The impetus here is both humane and practical: retaining experienced female staff by helping them cope with menopausal challenges. The broader impact is a reduction in stigma and a message that it’s acceptable to seek help and stay active during menopause
Online and peer support	United Arab Emirates	Formal programmes are rarer, but women are finding alternative avenues. One case is the use of online platforms and social media to foster exercise and discussion. In some Gulf countries, women-only Facebook groups or forums have sprung up to discuss menopause anonymously. These often share exercise tips, diet advice, and encouragement. For example, a participant from Saudi Arabia cited using a YouTube exercise channel at home as a key strategy. Additionally, emerging women's health groups in countries such as the UAE and Lebanon have started holding wellness workshops that include moderate exercise (such as group walks in malls or parks during women-only hours). Though not yet well documented, these small-scale efforts indicate a growing awareness. They also highlight how privacy and modesty concerns can be addressed by technology (virtual coaching) or by creating gender-segregated exercise opportunities
Regional networks and menopause societies	Malaysia, South Africa, and Turkey	Across these regions, professional societies are laying the groundwork that can include exercise promotion. The Asia Pacific Menopause Federation and national menopause societies in countries are conducting research and public seminars. Some have partnered with Ministries of Health to run community outreach—for instance, health camps where midlife women get screenings and learn about lifestyle management including exercise. While these efforts are in the early stages, they provide a platform for scaling up exercise-based interventions

Table 3 indicates gaps where not all women are reached (rural women, those outside formal employment, etc.), and programme sustainability can be an issue if reliant on volunteerism or short-term funding. Nonetheless, these cases demonstrate that the barriers to menopausal women exercising can be overcome with creativity and culturally conscious planning.

**Table 3:** Summary of exercise-related strategies for menopause support.

Strategy	Key actions	Cultural adaptation	Delivery mechanisms	Expected outcomes
Community-based exercise programmes	Local walking groups, dance classes, yoga/stretching sessions; led by trained facilitators; scheduled for convenience	Use culturally familiar activities (e.g. African dance and traditional music)	Community centres, outdoor spaces, peer-led groups	Peer support, reduced isolation, normalised menopause discussions
Women-only and culturally sensitive spaces	Safe venues in schools, religious institutions, or women’s clubs; privacy and modest attire respected	Align exercise with religious values (e.g. amanah in Islamic contexts)	Mobile health units, village health workers, after-hours use of facilities	Increased participation, culturally acceptable engagement
Integration into primary healthcare	Prescribe exercise during menopause care; provide toolkits in local languages	Sensitive counselling in line with local norms	Primary care, gynaecology clinics, routine check-ups	Normalise lifestyle intervention, improve symptom management
Leverage technology and media	WhatsApp/SMS tips, radio shows, online classes, YouTube	Culturally adapted digital fitness content	Government-NGO partnerships, mobile distribution	Increased reach, motivation for home-based exercise
Incorporate cultural practices	Use yoga, meditation, dance, gardening, courtyard walking	Embed in local traditions/spiritual practices	Community events, home-based routines	Greater acceptance, sustainability of habits
Policy and advocacy	National guidelines, awareness campaigns, workplace wellness programmes	Local role models, culturally relevant messages	Government, WHO, UNFPA frameworks	Structural change, workforce retention, public awareness
Inclusivity for all menopause types	Target surgical/medical menopause; adapt intensity	Mention explicitly in programmes and materials	Clinical follow-up, tailored support groups	Reduced exclusion, equitable symptom management

### Integrating exercise into menopause care: strategies and recommendations

To improve menopause management for women in Asia, Africa, and the Middle East, it is crucial to adopt pragmatic, cost-effective, and sustainable approaches that embed exercise into care in culturally appropriate ways. Below are key strategies, informed by the evidence and contexts discussed:

Exercise interventions for menopausal women should be community-based, culturally sensitive, and inclusive of all menopause types, including surgical and medically induced cases. Safe, women-only spaces and culturally adapted activities such as African dance, yoga, or courtyard walking can increase participation, especially in conservative settings. Integration into primary healthcare allows clinicians to prescribe and promote physical activity, supported by toolkits and culturally relevant resources. Technology and media, from SMS reminders to online video content, expand reach and sustain engagement, particularly for women in remote or resource-limited areas. Policy and advocacy efforts must formalise exercise promotion in national health strategies, ensuring structural support, workplace accommodations, and recognition of menopause as a public health and human rights priority (Figure 1).

## Conclusion

Exercise is a powerful implement in the menopausal toolkit—improving physical symptoms, bolstering brain health, and empowering women to take charge of their well-being. For women in Asia, Africa, and the Middle East, maximising this tool requires understanding and respecting cultural contexts. The evidence base shows that regular physical activity can alleviate depression, anxiety, insomnia, fatigue, and protect long-term cognitive health​. The sociological lens reminds us that a one-size approach will not fit all; programmes must negotiate gender norms, modesty, and differing attitudes towards menopause. Current efforts may offer blueprints for progress, although more culturally sensitive research on building effective exercise programmes is needed, which can help fill policy voids and healthcare gaps. By implementing community-grounded and culturally sensitive strategies, stakeholders, healthcare professionals, researchers, and policymakers can help women in these regions navigate menopause with greater ease and dignity. Integrating exercise into menopause care is not just about mitigating symptoms; it is about affirming women's agency and quality of life in the post-reproductive years. In every village, city, and society, a woman's midlife can be made healthier and happier by the simple act of moving her body, and it is time for our health systems to move with women.

## Data Availability

The original contributions presented in the study are included in the article/Supplementary Material; further inquiries can be directed to the corresponding author.

## References

[1] XuH LiuJ LiP LiangY. Effects of mind-body exercise on perimenopausal and postmenopausal women: a systematic review and meta-analysis. Menopause. (2024) 31(5):457–67. 10.1097/GME.000000000000233638669625 PMC11465887

[2] Economic Do. World Population Prospects 2024: Summary of Results. New York: Stylus Publishing, LLC (2024).

[3] UtianWH. Psychosocial and socioeconomic burden of vasomotor symptoms in menopause: a comprehensive review. Health Qual Life Outcomes. (2005) 3(1):47. 10.1186/1477-7525-3-4716083502 PMC1190205

[4] Eriksson BergmanL MatikasA LiuX FoukakisT. Adherence to adjuvant endocrine therapy including GnRH-analogues and survival: a population-based cohort study. eClinicalMedicine. (2025) 88:103493. 10.1016/j.eclinm.2025.10349341181852 PMC12572808

[5] Bagga SS Tayade S Lohiya N Tyagi A Chauhan A. Menopause dynamics: from symptoms to quality of life, unraveling the complexities of the hormonal shift. Multidiscip Rev. (2025) 8(2):2025057. 10.31893/multirev.2025057

[6] Santoro N Roeca C Peters BA Neal-Perry G. The menopause transition: signs, symptoms, and management options. J Clin Endocrinol Metab. (2021) 106(1):1–15. 10.1210/clinem/dgaa764 33095879

[7] HarringtonRB HarveyN LarkinsS Redman-MacLarenM. Family planning in Pacific island countries and territories (PICTs): a scoping review. PLoS One. (2021) 16(8):e0255080. 10.1371/journal.pone.025508034351949 PMC8341522

[8] DelanerolleG PhiriP ElneilS TalaulikarV ElejeGU KareemR Menopause: a global health and wellbeing issue that needs urgent attention. Lancet Glob Health. (2025) 13(2):e196–e8. 10.1016/S2214-109X(24)00528-X39708829

[9] PathirajaV KurmiO TohT-H ElejeGU Tweneboah-KoduahF MbweleB Exploring the availability and acceptability of hormone replacement therapy in LMICs using insights of pharmacists (MARIE Sri Lanka WP2a). Sci Rep. (2025) 15(1):37944. 10.1038/s41598-025-18083-x41168204 PMC12575872

[10] DelanerolleG GhoshS BriggsP PhiriP TaylorJ PathirajaV A Perspective on Economic Barriers and Disparities to Access Hormone Replacement Therapy in Low and Middle-Income Countries (MARIE-WP2d). (2025).10.1038/s41598-026-49559-zPMC1328430242036422

[11] ThivelD TremblayA GeninPM PanahiS RivièreD DuclosM. Physical activity, inactivity, and sedentary behaviors: definitions and implications in occupational health. Front Public Health. (2018) 6:288. 10.3389/fpubh.2018.0028830345266 PMC6182813

[12] Tiller NB. Exercise and sport: definitions, classifications, and relevance to population health. In: Raleigh SM, editor. Epigenetics of Exercise and Sports. Torrance, CA: Elsevier (2021). p. 3–22.

[13] Dasso NA, editor, How is exercise different from physical activity? A concept analysis. Nurs Forum. (2019) 54:45–52. 10.1111/nuf.12296 30332516

[14] DelanerolleG PhiriP ElneilS. The “menopause” knockout; a science-backed therapeutically, and strategy for midlife wellness. Front Sports Act Living. (2025) 7:1682887. 10.3389/fspor.2025.168288741487941 PMC12756445

[15] MudaligeT PathirajaV DelanerolleG CavaliniH WuS TaylorJ Systematic review and meta-analysis of the pelvic organ prolapse and vaginal prolapse among the global population. BJUI Compass. (2025) 6(1):e464. 10.1002/bco2.46439877583 PMC11771496

[16] Guerrero-González C Cueto-Ureña C Cantón-Habas V Ramírez-Expósito MJ Martínez-Martos JM. Healthy aging in menopause: prevention of cognitive decline, depression and dementia through physical exercise. Physiologia. (2024) 4(1):115–38. 10.3390/physiologia4010007

[17] HanB DuanY ZhangP ZengL PiP ChenJ Effects of exercise on depression and anxiety in postmenopausal women: a pairwise and network meta-analysis of randomized controlled trials. BMC Public Health. (2024) 24(1):1816. 10.1186/s12889-024-19348-238977980 PMC11229230

[18] Mitra S. From Evolution to Empowerment: A Holistic Framework for Menopause Management. Available at SSRN 5005927. (2024).

[19] Varalakshmi D Rekha K. The impact of exercise on glycemic control, cardiorespiratory, BMI and quality of life in postmenopausal diabetic women: a comprehensive review. Romanian J Diabetes Nutr Metab Dis. (2024) 31(1):82–92. 10.46389/rjd-2024-0082

[20] Lawson S Sacks A. Pelvic floor physical therapy and women’s health promotion. J Midwifery Women’s Health. (2018) 63(4):410–7. 10.1111/jmwh.12736 29778086

[21] Franco MM Pena CC de Freitas LM Antônio FI Lara LA Ferreira CHJ. Pelvic floor muscle training effect in sexual function in postmenopausal women: a randomized controlled trial. J Sex Med. (2021) 18(7):1236–44. 10.1016/j.jsxm.2021.05.005 34187758

[22] Sun Z Chen H Berger M Zhang L Guo H Huang Y. Effects of tai chi exercise on bone health in perimenopausal and postmenopausal women: a systematic review and meta-analysis. Osteoporos Int. (2016) 27(10):2901–11. 10.1007/s00198-016-3626-3 27216996

[23] ZouP LuoY WyslobickyM ShaikhH AlamA WangW Menopausal experiences of South Asian immigrant women: a scoping review. Menopause. (2022) 29(3):360–71. 10.1097/GME.000000000000191935213522

[24] ImEO KoY HwangH CheeW StuifbergenA LeeH Asian American midlife women’s attitudes toward physical activity. J Obstet Gynecol Neonatal Nurs. (2012) 41(5):650–8. 10.1111/j.1552-6909.2012.01392.x22789126 PMC3496028

[25] Darko N. South Asian and BME migrant women’s experiences of culturally tailored, women-only physical activity programme for improving participation, social isolation and wellbeing. In: Engaging Black and Minority Ethnic Groups in Health Research. Bristol: Policy Press (2021). p. 93–106.

[26] Shorey S Ang L Lau Y. Efficacy of mind–body therapies and exercise-based interventions on menopausal-related outcomes among Asian perimenopause women: a systematic review, meta-analysis, and synthesis without a meta-analysis. J Adv Nurs. (2020) 76(5):1098–110. 10.1111/jan.14304 31950541

[27] Shorey S Ng ED. The experiences and needs of Asian women experiencing menopausal symptoms: a meta-synthesis. Menopause. (2019) 26(5):557–69. 10.1097/GME.0000000000001269 30562319

[28] MeetaM DigumartiL AgarwalN VazeN ShahR MalikS. Clinical practice guidelines on menopause: *an executive summary and recommendations: Indian Menopause Society 2019–2020. J Midlife Health. (2020) 11(2):55–95. 10.4103/jmh.JMH_137_2033281418 PMC7688016

[29] LiQ GuJ HuangJ ZhaoP LuoC. “They see me as mentally ill”: the stigmatization experiences of Chinese menopausal women in the family. BMC Women’s Health. (2023) 23(1):185. 10.1186/s12905-023-02350-y37076835 PMC10116657

[30] Matina SS Mendenhall E Cohen E. Women’s experiences of menopause: a qualitative study among women in Soweto, South Africa. Glob Public Health. (2024) 19(1):2326013. 10.1080/17441692.2024.2326013 38497205

[31] DrewS KhutsoaneK BuwuN GregsonCL MicklesfieldLK FerrandRA Improving experiences of the menopause for women in Zimbabwe and South Africa: co-producing an information resource. Soc Sci. (2022) 11(4):143. 10.3390/socsci11040143

[32] Alidou S Verpoorten M. Only women can whisper to gods: voodoo, menopause and women’s autonomy. World Dev. (2019) 119:40–54. 10.1016/j.worlddev.2019.03.005

[33] Mensah BonsuI MyezwaH BrandtC AjidahunAT MosesMO AsamoahB. An exploratory study on excess weight gain: experiences of postmenopausal women in Ghana. PLoS One. (2023) 18(1):e0278935. 10.1371/journal.pone.027893536638076 PMC9838829

[34] Kyomuhendo C. Managing Menopause in the Workplace: Strategies for Professional Success and Support in Ugandan Higher Institutions of Learning. Available online at SSRN 4979872. (2024).

[35] Casas X. How the 'Green Wave' movement did the unthinkable in Latin America. International New York Times. (2021).

[36] ChaabaneS ChaabnaK DoraiswamyS MamtaniR CheemaS. Barriers and facilitators associated with physical activity in the Middle East and North Africa region: a systematic overview. Int J Environ Res Public Health. (2021) 18(4):1647. 10.3390/ijerph1804164733572229 PMC7914747

[37] Mustafa M Zaman KT Ahmad T Batool A Ghazali M Ahmed N. Religion and women’s intimate health: towards an inclusive approach to healthcare. In: Proceedings of the 2021 CHI Conference on Human Factors in Computing Systems. Yokohama: Association for Computing Machinery (2021). p. 1–13.

[38] ShahzadD ThakurAA KidwaiS ShaikhHO AlSuwaidiAO AlOtaibiAF Women’s knowledge and awareness on menopause symptoms and its treatment options remains inadequate: a report from the United Arab Emirates. Menopause. (2021) 28(8):918–27. 10.1097/GME.000000000000178333973540

[39] QutobRA AlaryniA AlsolamyEN Al HarbiK AlammariY AlanaziA Attitude, practices, and barriers to menopausal hormone therapy among physicians in Saudi Arabia. Cureus. (2024) 16(1):e52049. 10.7759/cureus.5204938344533 PMC10857802

